# A scoping review of current trends of multisectoral collaborations for health within the education, agriculture, and environment sectors in Anglophone Africa

**DOI:** 10.3389/fpubh.2025.1717941

**Published:** 2025-11-26

**Authors:** Ugenyi Victoria Iloabachie, Maureen Nwokorie, Casmir Mbaegbu, Kingsley Ude, Chinyere Ojiugo Mbachu, Obinna Onwujekwe

**Affiliations:** 1 Health Policy Research Group, College of Medicine, University of Nigeria, Enugu, Nigeria; 2 Department of Community Medicine, University of Nigeria Teaching Hospital, Enugu, Nigeria; 3 Department of Political Science, Faculty of the Social Sciences, University of Nigeria, Nsukka, Nigeria; 4 Department of Agricultural Economics, Faculty of Agricultural Sciences, University of Nigeria, Nsukka, Nigeria; 5 Department of Health Administration and Management, College of Medicine, University of Nigeria, Enugu, Nigeria

**Keywords:** multisectoral collaboration, social determinants of health, mainstreaming health, communicable diseases, non-communicable diseases, health-related policy, urban area, Anglophone Africa

## Abstract

**Background:**

In Anglophone Africa, addressing the double disease burden of disease requires coordinated action across both the health and non-health sectors. Despite the existence of policies advocating this multisectoral collaborations, implementation remains limited and inconsistent across the continent. This scoping review explored multisectoral collaboration for health in the education, agriculture and environment sectors in Anglophone Africa in order to understand the existence and effectiveness of policies and activities within these sectors that contribute to the prevention and control of communicable and non-communicable diseases.

**Methods:**

A scoping review was conducted across peer-reviewed and grey literature published between 2004 and 2024. It was guided by the Joanna Briggs Institute (JBI) framework and the Preferred Reporting Items for Systematic Reviews and Meta-Analyses (PRISMA) checklist, Databases such as HINARI, Google Scholar, PubMed, and African Journals Online (AJOL) were searched systematically. Search terms were customized for each database. Studies were included if they described or analyzed national or subnational health-related policies, programs, or initiatives involving any of these sectors in any Anglophone African country. The PAGER (pattern, advances, gaps, evidence and recommendations) framework was used to organize the results and streamline the presentation of data.

**Results:**

The review included 25 documents from Anglophone African countries. The findings show that while policies supporting multisectoral collaboration for health were identified, implementation remained inadequate. Key enablers of progress in multisectoral collaboration in mainstreaming health in the different sectors include strong political will, joint planning mechanisms, and donor coordination, while some barriers include poor inter-sectoral coordination, weak accountability frameworks, and limited data sharing.

**Conclusion:**

Though evidence exists of policies supporting multisectoral collaboration for health in the non-health sectors in anglophone Africa, translating policy intent into practice remains challenging. Strengthening governance, fostering institutional capacity, and promoting evidence-informed planning are critical in successful mainstreaming of health in this region.

## Introduction

The emergence and acceleration of non-communicable diseases (NCDs) in Africa has been rapid resulting in many countries being saddled with the coexistence of both communicable (CDs) (1). The simultaneous prevalence of NCDs and CDs within a given population is referred to as the double burden of disease (2) and is disproportionately pronounced in sub-Saharan Africa (3).

The double burden of disease places considerable strain on already fragile and under-resourced health systems with the resultant poor health conditions of the citizens (4). Historically, these English-speaking African countries have prioritized the control of CDs, which though still prevalent are now being accompanied by NCDs. According to the World Health Organization (WHO) Non-Communicable Disease Progress Monitor NCD-related mortality in Anglophone Africa ranges from 25% in Somalia to 81% in Mauritius (5) and possibly projected rise by the year 2030 if no effective intervention is implemented (6).

The social determinants of health such as food insecurity, poor sanitation, and limited health education exacerbate this health problem (7). Rapid urbanization in metropolitan cities such as Lagos, Accra, and Nairobi has fostered sedentary lifestyles, poor diet habits, and heightened exposure to air pollution which drive the increasing prevalence of NCDs (8).

The Commission of the Social Determinants of Health (CSDH) emphasize that non-health sectors can profoundly impact health outcomes and that addressing these sectors can lead to significant population health gains (9). In many LMICs, health systems are underperforming, leaving many population groups unable to access the care they require (10). In response, the WHO released new guidance advocating countries to systematically monitor social determinants of health (SDH) using integrated data systems, multisectoral collaboration, and enhanced political accountability as organizational frameworks (11).

Mainstreaming health into non-health sectors through multisectoral collaboration is not a new concept. The 1978 Declaration of Alma Ata initially introduced this concept, which was further reinforced in the 1989 Ottawa Charter for Health promotion, calling for the implementation of “health public policies” (12). Health in all policies (HiAP), vis a viz. multisectoral collaboration for health has received the highest political commitment globally, with the various WHO secretary generals endorsing it (13).

Mainstreaming health into non-health sectors is achieved by multisectoral collaboration actions between the health and non-health sectors (14). Though already being effectively practiced in the global north, the global south, where the majority of the low-and-middle-income countries are located is still struggling with the development and implementation of appropriate policies (15). The successful development and implementation of policies, activities and interventions for mainstreaming health into non-health sectors that require cooperation between the different non-health sectors and agencies is hinged on efficient governance (16).

There is limited literature available that explores multisectoral collaboration within the non-health sectors as a means of preventing and controlling CDs and NCDs in anglophone Africa (17). The purpose of this review was to explore the existing policies, and activities for mainstreaming health in the education, agriculture and environment sectors in the prevention and control of priority NCDs and CDs in the health sector and non-health sectors.

This review provides a better understanding of the landscape of multisectoral collaboration for health in anglophone Africa and offers recommendations for enhancing these collaborations to improve public health outcomes.

### Objectives

This review sought to explore the patterns of multisectoral collaboration for health in the agriculture, education, and environment sectors in Anglophone Africa. Taking into consideration the governance and finance models that have strengthened these efforts, while still highlighting gaps in coordination and accountability, reviewing the evidence of effectiveness, and outlining recommendations for assessing the long-term impact of multisectoral collaboration for health on priority communicable and non-communicable diseases.

## Methods

### Study design

This scoping review was guided by the Joanna Briggs Institute (JBI) methodology for scoping reviews. A scoping review process has been chosen because it allows the combination of data related to the project which has not previously been compiled in this manner (18, 19) and supports the identification of research gaps (20). The entire review process is reported in accordance with the Preferred Reporting Items for Systematic Reviews and Meta-Analyses extension for scoping reviews (PRISMA-ScR) protocol for scoping reviews (21).

#### Search strategy

The search strategy for this study involved a comprehensive literature search across multiple databases and relevant websites to identify evidence on health integration within the education, environment, and agriculture sectors in urban Anglophone Africa.

Databases such as HINARI, Google Scholar, PubMed, and African Journals Online (AJOL) were used to retrieve peer-reviewed articles, policy documents, and technical reports.

In total, 5,904 records were retrieved from these sources, including HINARI (17), Google Scholar (317), PubMed (5,561), and AJOL (9), ensuring a diverse and comprehensive pool of evidence for the review.

#### Boolean operators and search strings

This was *“multisectoral collaboration*,” AND (“health mainstream*” OR HiAP OR HIAP) AND (“communicable diseases” OR “infectious disease”) AND (“non-communicable diseases” OR “non-infectious disease”) AND (“Nigeria” OR “sub-Saharan Africa” OR “Anglophone Africa” OR Botswana OR Burundi OR Cameroon OR Eritrea OR Gambia OR Ghana OR Kenya OR Lesotho OR Liberia OR Madagascar OR Malawi OR Mauritius OR Namibia OR Nigeria OR Rwanda OR Seychelles OR Sierra Leone OR Somalia OR South Africa OR South Sudan OR Sudan OR Swaziland OR Tanzania OR Uganda OR Zambia OR Zimbabwe) AND “education sector,” “agriculture sector,” and “environment sector,” were used in the search process.

A standardized data extraction tool was designed in keeping with the Joanna Briggs Institute (JBI) methodology for scoping reviews (Supplementary Table 1) to ensure consistency and transparency in capturing the relevant information from all the included articles. This tool was pre-tested on a small sample of documents (six articles) and adjusted to ensure it aligned with the objectives of the review. The tool included columns for the extraction of data on author’s name(s), year of publication, source of article (web page or database), country, objectives, and key findings.

**Figure 1:**
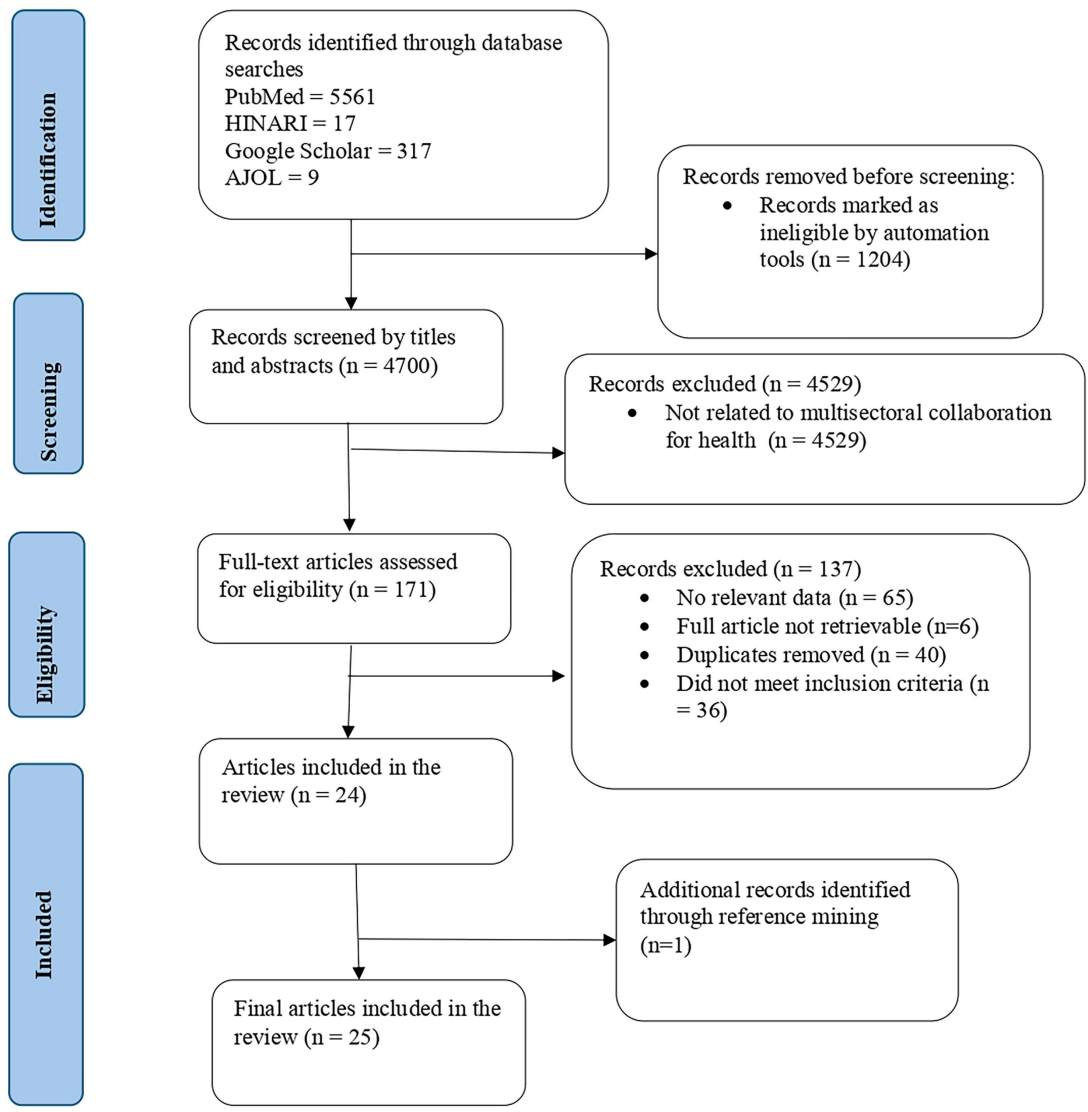
PRISMA-ScR diagram of source selection process for the review.

From the 5,904 extracted articles, a total of 1,204 that did not meet the inclusion criteria were excluded by respective search engines automation tools. Thereafter, the title and abstract screening was carried out by 4 members of the team, and the resulting 171 documents were entered into full text review. Reasons for exclusion of articles at full text were raised by the reviewers and any discrepancies were resolved through discussions within the team. The results of the search and the study inclusion process are presented in the PRISMA-ScR flow diagram as illustrated in Figure 1.

### Eligibility criteria

Studies were eligible if they were published in English language between 2004 and 2024, focused on Anglophone African countries, and examined multisectoral collaboration for health involving non-health sectors, specifically the education, agriculture, and environment sectors. Peer-reviewed journal articles, policy reports, and credible grey literature were included in the review. Studies were excluded if they focused exclusively on the health sector, were published in a language other than English or before 2004.

### Literature synthesis

Data was analyzed using the PAGER Framework (pattern, advances, gaps, evidence for practice and research recommendations). This strategy supported the research team to streamline the presentation of complex data, i.e., making the main result easier to understand.

The findings are organized using the PAGER framework’s thematic matrices.

The extracted data was analyzed thematically to provide a narrative synthesis of the current state of multisectoral collaboration for health within the non-health sectors of English-speaking African countries.

## Findings

Figure 1, the PRISMA-ScR flow chart, outlines the source selection process for the scoping review. Twenty-five studies were included in this scoping review and are summarized in Table 1. These studies examined multisectoral collaboration for health in 13 Anglophone African countries, with a particular focus on the roles of the education, agriculture, and environment sectors in addressing priority communicable and non-communicable diseases. The evidence base comprised peer-reviewed articles, policy reports, and grey literature published between 2004 and 2024, highlighting both the progress made and the persistent gaps in coordination, governance, and resource mobilization. Together, these studies provided insights into patterns, effectiveness, and challenges of implementing multisectoral collaboration for health in Anglophone Africa.

**Table 1:** Included review articles.

S/No	Author (year)	Country(ies)	Title of research article
1	Boidin B. (2020) (11)	Ghana Sierra Leone Senegal South Africa (Sub-Saharan Africa)	The social determinants of health in Africa from a political economy perspective: an exploratory contribution.
2	Akselrod S. (2024) (12)	Nigeria	Multisectoral action to address noncommunicable diseases: lessons from three country case studies.
3	Mauti J. et al. (2019) (13)	Kenya	Kenya’s Health in All Policies strategy: A policy analysis using Kingdon’s multiple streams
4	Weimann A. (2021) (15)	Africa	Intersectoral action for addressing NCDs through the food environment: An analysis of NCD framing in global policies and its relevance for the African context.
5	Rasanathan K. et al. (2017) (16)	Kenya Malawi Uganda	Governing multisectoral action for health in low- and middle-income countries.
6	Juma PA., et al. (2018) (17)	Kenya Malawi Nigeria Cameroon South Africa	Multi-sectoral action in non-communicable disease prevention policy development in five African countries.
7	Mahlangu P. et al. (2018) (41)	South Africa	Multisectoral (in)action: toward effective mainstreaming of HIV in public sector departments in South Africa.
8	Abdalazim Mohamed (2023) (42)	Sudan	C(h)allanges of primary health care in Sudan and the role of the health system building blocks as contributing factors: Rethinking Primary Health Care in Sudan’s Journey to Universal Health Coverage.
9	Ssennyonjo A. (2022) (35)	Uganda	What are the tools available for the job? Coordination instruments at Uganda’s national government level and their implications for multisectoral action for health.
10	Nganabashaka J. et al. (2022) (43)	Rwanda	Population-Level Interventions Targeting Risk Factors for Hypertension and Diabetes in Rwanda: A Situational Analysis.
11	Abubakar I. et al. (2022) (44)	Nigeria	The Lancet Nigeria Commission: investing in health and the future of the nation.
12	Mukanu M. et al. (2017) (45)	Zambia	Responding to non-communicable diseases in Zambia: a policy analysis.
13	World Health Organization (2013) (29)	Botswana Kenya Lesotho Rwanda Tanzania	Health in All Policies: report on perspectives and intersectoral actions in the African region.
14	Perez Arredondo A. et al. (2021) (46)	Ghana	Intersectoral collaboration shaping One Health in the policy agenda: A comparative analysis of Ghana and India.
15	Cairney P. et al. (2021) (47)	Kenya	The future of public health policymaking after COVID-19: a qualitative systematic review of lessons from Health in All Policies.
16	Abiona O. et al. (2019) (34)	Nigeria	Analysis of alcohol policy in Nigeria: Multi-sectoral action and the integration of the WHO “best-buy” interventions.
17	Benson T. (2011) (48)	Uganda	Cross-sectoral coordination in the public sector: A challenge to leveraging agriculture for improving nutrition and health.
18	Benson T (2007) (36)	Nigeria Uganda Ghana	Cross-sectoral coordination failure: How significant a constraint in national efforts to tackle ma in Africa?
19	Thondoo M. et al. (2024) (49)	Kenya South Africa	Multisectoral interventions for urban health in Africa: a mixed-methods systematic review.
20	Mapa-Tassou C. (2018) (50)	Cameroon	Two decades of tobacco use prevention and control policies in Cameroon: results from the analysis of non-communicable disease prevention policies in Africa.
21	Oluwasanu M. (2020) (51)	Nigeria	Multisectoral approach and WHO ‘Best buys’ in Nigeria’s nutrition and physical activity policies.
22	World Health Organization (2023) (52)	Africa (not specific)	Working Together for Equity and Healthier Populations: Sustainable Multisectoral Collaboration Based on Health in All Policies Approaches.
23	Nyaaba G. et al. (2020) (37)	Ghana	Implementing a national non-communicable disease policy in sub-Saharan Africa: Experiences of key stakeholders in Ghana.
24	Bennett S. et al. (2018) (53)	Nigeria	Governing multisectoral action for health in low-income and middle-income countries: unpacking the problem and rising to the challenge.
25	Amuyunzu-Nyamingo M. (2010) (54)	Kenya	Need for a multi-factorial, multi-sectorial and multi-disciplinary approach to NCD prevention and control in Africa.

**Figure 2:**
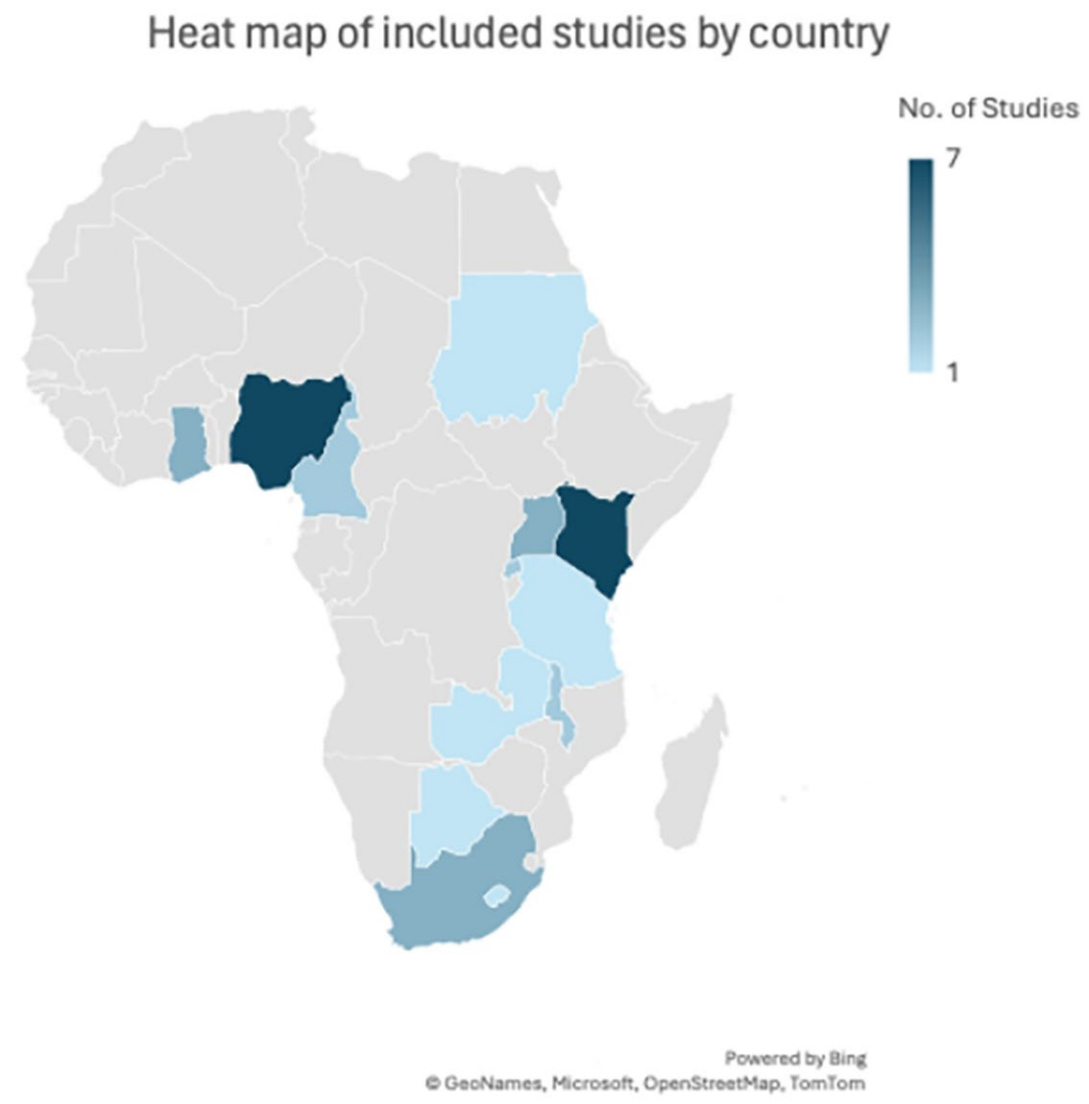
Distribution of included review articles by country.

The map below, Figure 2, shows the distribution of all the studies included in the review except the studies which did not have any particular focus country but were generalized for the African continent (11, 15).

**Figure 3:**
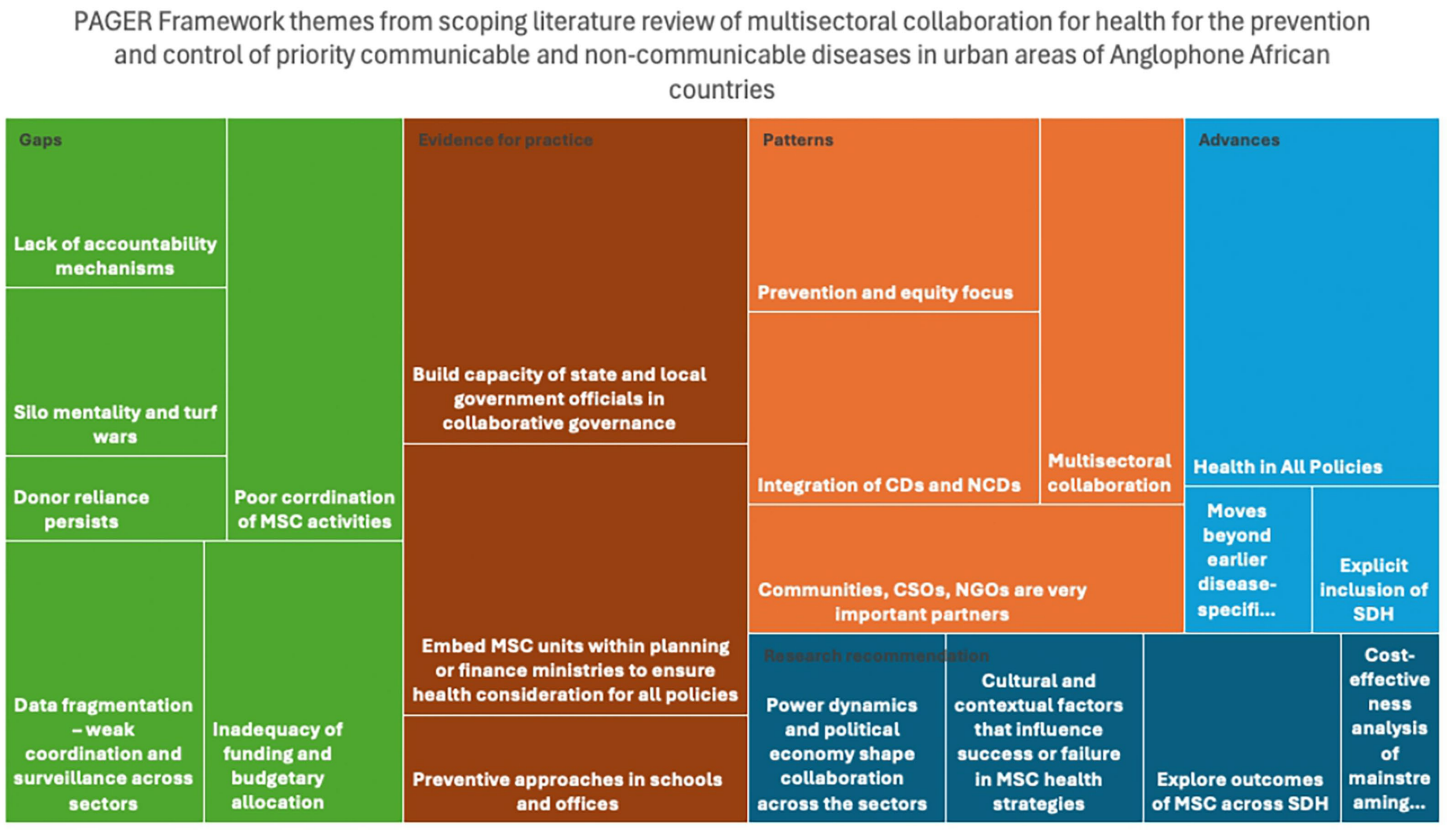
Summary of themes generated applying the PAGER framework.

This review applied the PAGER framework (patterns, advances, gaps, evidence for practice and research recommendations) (22), as shown in Table 2, to systematically analyze and organize the findings (Figure 3). Within the education, agriculture and environment sectors that focused on the prevention and control of communicable and non-communicable diseases in Anglophone African countries.

**Table 2:** Key findings using PAGER framework.

Themes	Sectors involved	Patterns	Advances	Gaps	Evidence for practice	Recommendations
Government-based multisectoral collaboration addressing CDs and NCDs.	Health, Agriculture, Education, Environment	Emphasis on multisectoral collaboration, recognizing that health outcomes depend on non-health sectors as well (11–13, 15, 17, 41, 42, 44, 47, 48, 52–54). Recurrent focus on health system strengthening, community engagement, and equity in access (11, 12, 17, 42, 52, 54). Some non-health sectors still perceive diseases as a health issue, which was outside their mandate (41, 47, 52, 53). Communities, civil society groups and non-governmental organizations, are very important partners in multisectoral activities (15, 34, 42, 43, 49, 52–54). Ministries of Health often lead coordination (16, 17, 42, 47, 53).	Moves beyond earlier disease-specific vertical programs by creating a unified framework for both CDs and NCDs (42, 53). ‘Health in All Policies’ approach—aligns national development with disease prevention goals (11–13, 29, 34, 42, 44, 47, 52–54). Advances in collaborative policies, e.g., integration of NCD risk factors (tobacco, alcohol, unhealthy diets, physical inactivity) with infectious disease control (12, 13, 29, 34, 41, 42, 52–54). Explicit inclusion of social determinants of health such as poverty, education, urbanization, and environment (11, 13, 29, 34, 42, 47, 48, 52, 54). Introduction of monitoring and evaluation frameworks with national and subnational accountability (42, 52, 53). Growing global recognition of need for MSC – WHO HiAP manuals (11–13, 42, 52, 53).	Poor coordination mechanism at the State and Local Government levels (11, 17, 35, 37, 42, 44, 47, 48, 50, 51, 53, 54). Working in silos between the different sectors (16, 35, 41, 42, 52). HiAP is seen predominantly as a health sector effort, with limited intersectoral cooperation (11, 13, 46, 47). Inadequacy of funding and budgetary allocation (3, 11, 17, 36, 42, 43, 45, 48, 53, 839). Low level of awareness by various sectors about their potential contribution, poor political will, coordination complexities and inadequate resources (13, 17, 35, 37, 42, 43, 47, 52, 53). Inadequate clarity on roles and responsibilities between sectors, causes an overlap causing competition (12, 16, 17, 35, 37, 42, 43). Gaps in communication, coordination, data quality and monitoring for better policy planning (42, 43, 48, 52). Data fragmentation – weak coordination and surveillance across sectors (11, 16, 35, 37). Lack of appropriate accountability and transparency mechanisms (11, 12, 35, 52).	Preventive approaches such as vaccination, health promotion, lifestyle modification, sanitation, and nutrition (13, 41, 43, 52). Embed MSC units within budget and planning or finance ministries to ensure health consideration for all policies, e.g., in the response to HIV and AIDS in South Africa (41, 52). The establishment of a well-funded, efficient and multi-disciplinary committee (16, 29, 43, 51, 52).	Integration of communicable and non-communicable disease responses under a single plan, to avoid siloed approaches (29, 41, 42, 44). Comparative studies on effectiveness of community-led vs. top-down models (11, 29, 41, 47). Explore models of pooled funding and inter-sector accountability (12, 29, 44). Study how power dynamics and political economy shape collaboration across the sectors (12, 17, 29, 35, 42, 44, 46, 47, 53). Explore outcomes of multisectoral collaboration for health across social determinants of health (11, 17, 29, 44, 47). Evaluate cost-effectiveness analysis of mainstreaming health in all policy models in anglophone Africa (13). Evaluate long-term impact of mainstreaming health in all policy models in anglophone Africa (13, 35). Explore cultural and contextual factors that influence success or failure in MSC health strategies (12, 35). Need for a multisector committee on hygiene to drive preventive efforts (44).
Agriculture sector-specific collaborations		Involved in nutrition and food security activities/initiatives (15, 17, 35, 42). School feeding programs (29).	Growth of multisectoral nutrition policies (e.g., Nutrition programs in Nigeria, Uganda, Rwanda, Ghana) (15).	Few evaluations of long-term health outcomes (15). Limited collaboration at local government level (48).	Food-based interventions for tackling malnutrition (15).	Examine climate change adaptation the links to nutrition and health.
Education sector-specific linkages		Schools are utilized as platforms for health promotion exercises (health education, nutrition) (42, 50, 54). Teachers are usually engaged as health educators (42).	Integration of age-appropriate health education in the curriculum (54).	Limited data on scale-up of successful school health interventions.		
Environmental sector-specific collaboration		Partnerships are mainly around environmental sanitation and vector control and management (13, 42).		Lack of scientific evidence on climate change and collaboration.		

### Success stories of multisectoral collaboration for health in Anglophone Africa

#### Education sector

##### Kenya’s School Health Policy

Kenya developed a National School Health Policy in 2009 (23) and a second edition in 2018 (24), to provide health education, improve sanitation in schools, and promote adolescent reproductive health. The program resulted in improved knowledge of hygiene and reduced absenteeism due to illness.

##### Nigeria’s Renewed Hope, National Home-Grown School Feeding Program

Nigeria’s initiative established in 2005, provides free nutritious meals to schoolchildren, sourced from local farmers. This has led to better child nutrition, increased school attendance, and economic benefits for smallholder farmers (25).

##### Uganda’s malaria smart schools

This project piloted in collaboration with USAID engages pupils and teachers as active agents in malaria prevention—integrating age-appropriate malaria education into the school curriculum and mobilizing pupils to adopt environmental control measures and use treated nets (26, 27).

#### Agriculture sector

##### Ethiopia’s Productive Safety Net Program

Launched in 2005, this initiative links food security with public health by providing cash or food to vulnerable households in exchange for community work (e.g., soil conservation, water harvesting) (28). It has improved nutrition and reduced food insecurity among low-income populations.

##### The Nutritional Improvement for Children in Urban Chaani in Kenya (NICK)

The project began in 2010 in response to the report of the World Health Organization Commission on Social Determinants (29). It involved forming an urban nutrition working group with membership spanning the health, education and environment sectors (30).

#### Environment sector

##### South Africa’s Climate and Health Plan

South Africa’s National Climate Change Response Policy of 2011, which set the stage for the National Climate Change and Health Adaptation Plan of 2014, integrate health considerations into climate action, addressing the links between air pollution, heat stress, and disease patterns. Policies focus on reducing emissions, improving public health resilience, and protecting vulnerable populations (31).

##### Uganda’s integrated malaria management

Uganda has combined environmental vector control, improved drainage, and insecticide-treated nets to reduce malaria transmission, particularly in flood-prone areas (32).

##### Kenya Tobacco Control Act

Through the efforts of the Ministry of Health and the civil society organizations, the Kenyan government was able to successfully ban smoking in public places in 2007 (33).

## Discussion

This scoping review shows that a very important aspect of operationalizing multisectoral collaboration (MSC) for health is the early engagement of the relevant stakeholders (12). MSCs are influenced by political debates and interests. It is therefore pertinent that policy makers and practitioners understand the politics and priorities of the other sectors to be able to explain what the collaborative effort hopes to achieve (28). National policies generally are ‘top down’ in focus and lack attention to the decentralization to state and local government service delivery levels (34).

Most of the countries reported insufficient financial resources allocated for engaging multiple sectors in MSC policy implementation activities (35–37). The main barriers to multi-sectoral action included lack of awareness by various sectors about their potential contribution, weak political will, coordination complexity and inadequate resources (17). Weak political will is a major consequence of power attrition (38). Frequent changes in political leadership, administrative reshuffling, and patronage-driven governance—continues to undermine sustained multisectoral collaboration for health (13, 39). Leadership turnover often disrupts continuity in health policies and weakens the institutional memory necessary to maintain multisectoral partnerships (40). Consistently high commitment from policymakers helps to keep multisectoral collaboration for health relevant and also helps cut through ‘administrative silos‘and address ‘turf wars’. Conversely, low or fleeting commitment is a major cause of poor implementation (37).

Another key approach to multisectoral collaboration from this review was the framing or re-framing of CDs and NCDs, such that they are not seen just as a problem of the health sector alone (3). In South Africa, HIV was re-framed, as a developmental challenge impacting on educational outcomes as well as recognizing that it does not only affect individuals but entire families (3). These framings facilitated the commitment and buy-in of the non-health sectors (3). These non-health sectors recognizing the impact of the human immunodeficiency virus (HIV) infection, began integrating HIV in their programs and budgets, while still tailoring the response in line with their core mandate and comparative advantage (41).

In conclusion, the evidence gathered from the scoping review demonstrate that national policies, activities and programs aimed at multisectoral collaboration for health with the non-health sectors are existent, though not fully operational or efficient in most cases. The analysis revealed several consistent patterns across the included studies. These patterns reflect recurring challenges, common facilitators, and thematic overlaps that characterize the existing evidence base such as prevention and social determinants of health focus as well as importance of the inclusion of communities, CSOs and NGOs.

In addition to recurring trends, notable advances were identified. These advances represent instances of innovation, emerging best practices, and methodological or policy shifts that mark progress with multisectoral collaboration for health in the African continent.

Despite these advances, critical gaps persist. These gaps manifest in structural issues, insufficient funding, and poor accountability mechanisms. Beyond these descriptive findings, the literature review synthesis also generated insights that bear direct implications for policy and practice. The evidence generated highlights strategies that are scalable, and adaptable in the local context.

Finally, the review was able to highlight some priority areas for future research. These recommendations reiterate the importance of addressing identified gaps, adopting more rigorous and context-sensitive methodologies, and testing innovative models to strengthen both academic knowledge and applied practice. Such directions are critical for advancement and for ensuring that subsequent research responds to real-world needs.

There is therefore need to establish integrated governance structures, and context-specific strategies aimed at the prevention and control of communicable and noncommunicable diseases while paying strict attention to the social determinants of health.

### Limitations

A major limitation of this review was inclusion of only articles published in English language. This means that any articles published in other languages as well as regional reports published in non-English outlets that may have contained vital information for the review were excluded.

Also, articles pertaining to multisectoral collaboration for health was only available for 13 of the 16 anglophone countries we intended to study for the purpose of this review, thus limiting the generalizability of findings across the entire sub-group. This absence of data from some countries may be as a result of gaps in publication, limitation in research capacity, or restrictions of public access to national policy documents.

Also, the search strategy and databases used to conduct searches; despite efforts to design comprehensive search strings, differences in indexing terms and structures of keywords on each respective database may have led to the omission of potentially relevant studies or policies.

Finally, as is common with all scoping reviews, the data synthesis was purely descriptive and did not include critical appraisals of the quality of the articles, which may affect the credibility of the evidence base.

## Data Availability

The original contributions presented in the study are included in the article/Supplementary material, further inquiries can be directed to the corresponding author.
